# US Food and Drug Administration Accelerated Approval Program for Nononcology Drug Indications Between 1992 and 2018

**DOI:** 10.1001/jamanetworkopen.2022.30973

**Published:** 2022-09-09

**Authors:** Kenji Omae, Akira Onishi, Ethan Sahker, Toshi A. Furukawa

**Affiliations:** 1 Department of Innovative Research and Education for Clinicians and Trainees, Fukushima Medical University Hospital, Fukushima, Japan; 2 Center for Innovative Research for Communities and Clinical Excellence, Fukushima Medical University, Fukushima, Japan; 3 Department of Advanced Medicine for Rheumatic Diseases, Kyoto University Graduate School of Medicine, Kyoto, Japan; 4 Department of Health Promotion and Human Behavior, Kyoto University Graduate School of Medicine, School of Public Health, Kyoto, Japan; 5 Population Health and Policy Research Unit, Medical Education Center, Kyoto University Graduate School of Medicine, Kyoto, Japan

## Abstract

**Importance:**

The US Food and Drug Administration (FDA) grants accelerated approval according to surrogate measures of numerous drug indications for serious or life-threatening illnesses such as infectious diseases and cancer. Investigators, including the FDA, have evaluated the program’s regulatory and clinical consequences in oncology, but evaluation of nononcology drugs is lacking.

**Objective:**

To evaluate the accelerated approval program for nononcology drug indications over a period of 26 years.

**Design, Setting, and Participants:**

This retrospective cohort study used publicly available data on FDA nononcology drug indications granted accelerated approval from June 1992 through May 2018, with preapproval and confirmatory trials for approved drugs. Data were analyzed from February to April 2022.

**Main Outcomes and Measures:**

The study estimated the median time from accelerated approval to occurrence of regulatory outcomes such as regular approval conversion, postapproval boxed warning label changes, confirmatory trial completion, and confirmatory trial results publication.

**Results:**

The FDA granted accelerated approval of 48 drugs for 57 nononcology indications, including 23 (40%) HIV treatments, supported by 93 preapproval trials. Forty-three indications (75%) were converted to regular approval at a median time of 53.1 (95% CI, 38.7 to 70.2) months from accelerated approval. There were postapproval label modifications on boxed warnings in 27 indications (47%) with a median time of 248.6 (95% CI, 51.8 to not estimable) months from accelerated approval. Of the 86 required confirmatory trials, 17 (20%) had not fulfilled the postapproval requirements. The median time to confirmatory trial completion was 39.4 (95% CI, 30.7 to 47.9) months. Nine trials (10%) failed to verify clinical efficacy, but only 1 of 8 indications assessed (2%) was withdrawn owing to the failed confirmatory trial, which was 136 months after approval. Results were published in 56 completed confirmatory trials (65%), with the median time being 52.5 (95% CI, 35.6 to 82.2) months from accelerated approval to publication.

**Conclusions and Relevance:**

Although the program expedited the approval of nononcology drug indications by a median (IQR) of 53.1 (26.8-133.2) months, safety-related label modifications were often added in boxed warnings after approval, and clinical efficacy was sometimes not confirmed. The study findings and long follow-up period suggest that comprehensive evaluation of such drugs may take more than a decade.

## Key Points

**Question:**

How has the US Food and Drug Administration (FDA) accelerated approval program been used in nononcology areas?

**Findings:**

In this cohort study, the FDA granted accelerated approval of 48 drugs for 57 nononcology indications from 1992 to 2018 with a median time to regular approval of 53.1 (95% CI, 38.7-70.2) months. Nine postapproval confirmatory trials failed to verify clinical efficacy, but only 1 indication was withdrawn due to a failed confirmatory trial 136 months after approval.

**Meaning:**

These findings suggest that the FDA accelerated approval program has provided access to expedited treatments for patients with severe nononcology diseases; however, a comprehensive drug evaluation will take more than a decade.

## Teaser

This cohort study evaluates how the US Food and Drug Administration (FDA) accelerated approval program has been used for nononcology drugs over a period of 26 years.

## Introduction

In 1992, Congress authorized the creation of the US Food and Drug Administration (FDA) accelerated approval program to address unmet medical needs via rapid drug development in the wake of the HIV/AIDS crisis.^1^ Under the program, drug approval can be based on surrogate measures deemed reasonably likely to project actual clinical end points (eg, symptom change and mortality), including intermediate clinical end points and biomarkers.^2^ Using surrogate measures in clinical trials can bring drugs to market faster than actual clinical end points. Instead, the FDA requires that manufacturers conduct postapproval trials to determine drug efficacy and risks.^3^ Current FDA guidelines require documented maintenance of a confirmatory trial during accelerated approval and an official label citing that clinical benefit has not been established. Upon trial completion, the label is revised and the indication is converted to regular approval with confirmation of clinical benefit. However, the drug may be removed from the market if FDA requirements are not met or if the trial fails to verify clinical benefit.^4^

Given the uncertainty regarding the predictability of actual meaningful clinical benefit through surrogate measures, accelerated approval has been controversial, particularly in oncology. The FDA recently reported on 25 years of the program. They examined regulatory consequences of 93 oncology indications granted accelerated approval between December 1992 and May 2017.^5^ They concluded that most oncology drugs approved via the program were eventually determined safe and effective in confirmatory trials. However, a different study^6^ found that a few drugs were determined to have verified benefits according to improvement in survival reported in confirmatory trials, and that confirmatory trials were sometimes substantially delayed or incomplete. In addition, the program may be associated with increased safety concerns in the postmarketing phase, including label modifications on boxed warnings and withdrawals.^7,8^ Payers, policy makers, and patients have expressed concerns regarding financial waste following approval of drugs eventually proving ineffective or unsafe.^9,10^ The most widely discussed example is bevacizumab for the treatment of metastatic breast cancer. Accelerated approval was granted in 2007 according to the progression-free survival observed in an open-label randomized clinical trial.^11^ However, confirmatory trials showed no improvement in overall survival and increased toxic effects, thereby prompting the FDA to withdraw approval in 2011.

To date, only a few studies have evaluated the program for nononcology indications. Such indications cover a fairly wide range of relevant therapeutic areas, such as emerging infectious diseases and progressive neuromuscular diseases, which lack reasonable treatment choices.^12^ Notably, aducanumab-avwa for the treatment of Alzheimer disease—the very recently approved nononcology indication relying on an unvalidated surrogate biomarker—has fueled the controversy of the program owing to its potential risks and high costs.^13,14^ To address this evidence gap, we reviewed and assessed nononcology drugs under the FDA’s accelerated approval program from its implementation in 1992 through 2018. We characterized preapproval and confirmatory trials, examining key regulatory outcomes including completion of confirmatory trials, publication of results, conversion to regular approval, and postapproval safety outcomes.

## Methods

### Eligible Drug Indications and Search Strategy

This retrospective cohort study was based on publicly available data involving no individual patient information and institutional review board approval was not required in accordance with 45 CFR §46. This study followed the Strengthening the Reporting of Observational Studies in Epidemiology (STROBE) reporting guideline. The protocol is available in medRxiv.^15^ To identify all nononcology drug indications granted accelerated approval between June 1992 and May 2018, we searched the Drugs@FDA database^16^ and reviewed publicly available documents at the Center for Drug Evaluation and Research^17^ and the FDA’s annual new drug summaries, following previous study procedures.^18,19,20^ We selected drugs that received accelerated approval as new therapeutic agents and as supplemental approvals (already approved for other indications). When there were simultaneous approvals for different formulations (eg, tablet and injection) of the same drug, only 1 approval was selected. The inclusion of drugs approved up to May 2018 allowed for at least 3.5 years for completion and publication of confirmatory trials.

### Identification of Preapproval and Confirmatory Trials and Data Extraction

We reviewed medical review reports and product labels for each drug indication available in the Drugs@FDA database to identify preapproval trials supporting accelerated approval. The medical review comprises integrated summaries of safety and efficacy and a description of relevant individual clinical trials. For confirmatory trial requirements, such as study design, target population, primary end point, due date, and trial identification number assigned by manufacturers, we searched the database of postmarket requirements and commitments on Drugs@FDA,^21^ as well as approval letters at the time of accelerated approval. If an indication had been converted to regular approval, we reviewed the approval letter and medical review at the time of regular approval to obtain completed confirmatory trial detailed information. We further conducted an internet search for associated press releases when no relevant documents were available on Drugs@FDA. In addition, we searched ClinicalTrials.gov using the drug name, indication, sponsor type, study design and phase, target population, and primary end point as search terms and identified the National Clinical Trial number assigned for each trial.

The following trial data were extracted: design (randomization and blinding), comparator (active, placebo, other, or none), participant enrollment, primary end points, and the start and end dates. End points were divided into clinical outcomes (patient-reported and performance outcomes) and biomarkers (histological, laboratory, magnetic, or physiological).^12,22,23^ For completed confirmatory trials, we determined if clinical efficacy was verified in trial results. One investigator (K.O.) searched relevant trials and another (A.O.) verified search results. Disagreements were resolved by consensus. If trials were unidentified, we contacted the manufacturers for clarification.

### Assessment of Regulatory Outcomes and Publication Matching

Conversion to regular approval and approval date were determined from the most recent drug label and associated letter published in the approval history section of Drugs@FDA. Postapproval requirement status was identified in the postmarket requirements and commitments in Drugs@FDA.^21^ This database categorizes the status as ongoing, pending, delayed, terminated, submitted, fulfilled, or released. ClinicalTrials.gov was then searched to verify the status of confirmatory trials (eg, still recruiting, ongoing but no longer recruiting, or completed) for each drug indication.

We also investigated 2 of the most clinically important postapproval safety-related events, withdrawal and additional boxed warnings on the label. The manufacturer or the FDA will withdraw the drug from the market when a new, potentially life-threatening safety issue associated with the drug profoundly changes the balance of risks and benefits. For each drug indication with discontinued marketing status in the Drugs@FDA database, we searched the associated approval letter and the *Federal Register* to determine whether safety was the discontinuation reason. If no relevant documents were available, an internet search for press releases involving the drug name and the term “discontinue” or “withdrawal” was conducted. A boxed warning is issued by the FDA when a serious safety risk has been detected and the risk-benefit balance favors continued use of the drug. To ascertain label modifications, we searched the MedWatch database available on Drugs@FDA in addition to the associated approval letter.^24^ For label changes that occurred between July 1996 and December 2007, we downloaded the archive file and extracted the records.^25^ For label changes from January 2008 to December 2015, we retrieved the data from monthly tables available on MedWatch and searched Drug Safety-related Labeling Changes for information after January 2016.^26^ We compared the revised label on the revision date with the most recent archived label for confirmation, and recorded the earliest date of the label update on boxed warnings.

We searched PubMed, Google, Google Scholar, and manufacturer websites to match each identified trial with publications in medical literature. Abstracts or full texts in all languages were reviewed. Trials were matched with publications according to trial characteristics (eg, National Clinical Trial number and/or trial identification, drug name, comparator, enrollment, dosing schedule, and primary end point). For 15 confirmatory trials related to 11 drug indications that remained unmatched to a publication, we contacted manufacturers to clarify the publication status. All the contacted manufacturers responded.

All searches were first made in June 2021 and last updated in January 2022. One investigator (K.O.) screened the databases, and another (A.O. or E.S.) verified the results. All disagreements were resolved through discussion.

### Statistical Analysis

We descriptively analyzed FDA program use trends over time, the characteristics of preapproval and confirmatory trials, and the aforementioned regulatory outcomes. The World Health Organization’s Anatomic Therapeutic Classification system was used to classify drug indications into 1 of 14 different therapeutic areas: alimentary tract and metabolism; anti-infectives for systemic use; antineoplastic and immunomodulating agents; antiparasitic products, insecticides, and repellents; blood and blood-forming organs; cardiovascular system; dermatologicals; genitourinary system and sex hormones; musculoskeletal system; systemic hormonal preparations, excluding sex hormones and insulins; nervous system; respiratory system; sensory organs; and various other therapeutic areas.^27^ We further differentiated HIV anti-infectives from the others, as HIV-related therapies are the first and most common therapeutic areas other than oncology granted accelerated approval. Notably, the indication of treprostinil sodium approved for pulmonary arterial hypertension was classified as cardiovascular system instead of blood and blood-forming organs. The median time to event was estimated using the Kaplan-Meier method. For regular approval conversion, the first episode of additional boxed warning, and confirmatory trial completion, if the event of interest had not yet occurred, withdrawal as well as follow-up completion (January 31, 2022) were regarded as right-censoring. If the date of confirmatory trial completion was unclear, the earlier of the publication date or the date of regular approval conversion was substituted. We used STATA statistical software version 16 (Stata Corp LP) for the analysis. Data were analyzed from February to April 2022.

## Results

### Accelerated Approvals for Nononcology Drug Indications

From June 1992 through May 2018, the FDA granted accelerated approval of 48 drugs for 57 nononcology indications (eFigure and eTable in the Supplement). We identified 93 preapproval trials with 86 requiring postapproval confirmatory trials (eFigure in the Supplement). The approved indications included a wide range of therapeutic areas, with anti-infectives for HIV (23 approvals [40%]) and other infectious diseases (7 approvals [12%]) being the most common (Table 1). With the exception of 2009 and 2010, 1 to 6 indications were approved via the program each year (Figure). Indications for HIV treatments in particular were approved almost every year from the initial approval in 1992 to 2008.

**Table 1.** Characteristics and Regulatory Outcomes of Nononcology Drug Indications Approved by the US Food and Drug Administration

Characteristics or outcomes	Indications, No. (%)
Therapeutic area^a^	
Alimentary tract and metabolism	3 (5)
Anti-infectives for systemic use for HIV	23 (40)
Anti-infectives for systemic use for non-HIV	7 (12)
Antineoplastic and immunomodulating agents	3 (5)
Antiparasitic products, insecticides, and repellents	1 (2)
Blood and blood-forming organs	1 (2)
Cardiovascular system^b^	4 (7)
Dermatologicals	1 (2)
Genitourinary system and sex hormones	2 (4)
Musculoskeletal system	1 (2)
Systemic hormonal preparations, excluding sex hormones and insulins	1 (2)
Various	10 (18)
Approval type	
Novel	51 (89)
Supplemental	6 (11)
Time to accelerated approval, median (IQR), mo^c^	7.8 (6.0-10.9)
Conversion to regular approval	43 (75)
Estimated time to regular approval, median (95% CI), mo^d^	53.1 (38.7-70.2)
Withdrawal	4 (8)
Postapproval changes in boxed warning on labels	27 (47)
Estimated time to changes in boxed warning on labels, median (95% CI), mo^d^	248.6 (51.8-not estimable)

^a^ Therapeutic areas were classified according to the Anatomical Therapeutic Chemical classification system index 2022. We further differentiated anti-infectives for HIV from the others.

^b^ Indication of treprostinil sodium approved for the treatment of pulmonary arterial hypertension was classified as cardiovascular system instead of blood and blood-forming organs.

^c^ Time from the date when the FDA received an application from manufacturers.

^d^ Time from the date of accelerated approval.

**Figure.**
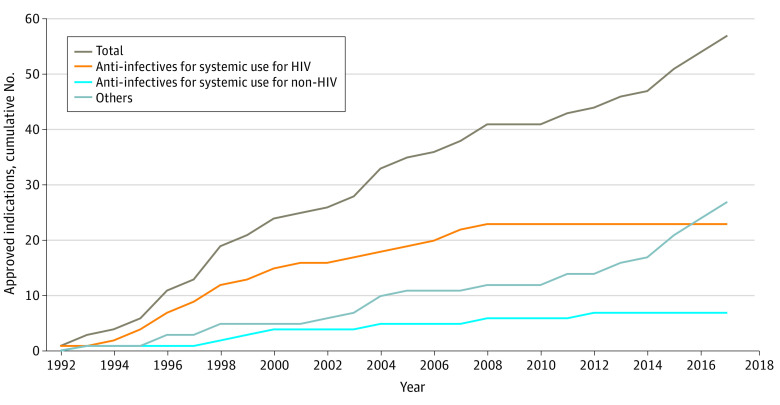
Cumulative Instances of Use of Accelerated Approval Program for Nononcology Drugs, June 1992 to May 2018

The median (IQR) time from the FDA's receipt of a manufacturer application to accelerated approval was 7.8 (6.0 to 10.9) months. The estimated median time from accelerated approval to regular approval conversion was 53.1 months (95% CI, 38.7 to 70.2 months), or approximately 4.5 years. Forty-three indications (75%) converted to regular approval. Four indications (8%) were withdrawn, 1 (dalfopristin/quinupristin for vancomycin-resistant *Enterococcus faecium*) by the FDA because the submitted data failed to verify clinical benefit, and the others (lutropin-α concomitantly administered with follitropin-α for stimulation of follicular development in infertile hypogonadotropic hypogonadal women with profound luteinizing hormone deficiency, and levofloxacin for the treatment of inhalational anthrax) by the manufacturers for reasons unrelated to safety or effectiveness. In 27 indications (47%), postapproval label modifications were added on boxed warnings with the estimated median time from accelerated approval being 248.6 (95% CI, 51.8 to not estimable) months and the shortest period being 5.0 months. There were no safety-related withdrawals.

### Features of Preapproval and Confirmatory Trials

The median (IQR) number of participants enrolled in the preapproval and confirmatory trials were 232 (97-449) and 453 (164-718), respectively (Table 2). Eighty-four (90%) preapproval trials and 68 (79%) confirmatory trials were randomized. Sixty-one (66%) preapproval trials and 46 (53%) confirmatory trials were double-blinded. Eight (9%) preapproval trials and 11 (13%) confirmatory trials had no comparators. Depending on the therapeutic area, different types of outcomes, such as biomarkers and clinical outcomes, were measured as primary end points in preapproval and confirmatory trials. In the therapeutic areas of antineoplastic and immunomodulating agents, cardiovascular system, dermatologicals, and genitourinary system or sex hormones, all primary end points were clinical in both preapproval and confirmatory trials. Alternatively, in the areas of antiparasitic products, insecticides and repellents, blood and blood-forming organs, and systemic hormonal preparations excluding sex hormones and insulins, no clinical outcomes were evaluated, even in confirmatory trials.

**Table 2.** Characteristics of Preapproval and Confirmatory Trials

Characteristics	Trials, No. (%)	
	Preapproval (n = 93)^a^	Confirmatory (n = 86)
Enrollment, median (IQR), No. of participants	232 (97-449)	453 (164-718)
Randomized	84 (90)	68 (79)
Double-blind	61 (66)	46 (53)
Comparator		
Placebo	55 (59)	39 (45)
Active	15 (16)	18 (21)
Add-on	2 (2)	3 (3)
Other	13 (14)	13 (15)
None	8 (9)	11 (13)
Unclear	NA	2 (2)
Primary end points used by therapeutic area^b^^,^^c^		
Alimentary tract and metabolism		
Clinical	1 (33)	1 (17)
Histologic	1 (33)	NA
Laboratory	1 (33)	1 (17)
Physiologic	NA	1 (17)
Anti-infectives for systemic use for HIV		
Clinical	2 (4)	16 (31)
Laboratory	47 (100)	39 (75)
Anti-infectives for systemic use for non-HIV		
Clinical	NA	2 (29)
Laboratory	7 (100)	4 (57)
Antineoplastic and immunomodulating agents, clinical	4 (100)	5 (100)
Antiparasitic products, insecticides, and repellents, laboratory	2 (100)	1 (100)
Blood and blood-forming organs, laboratory	1 (100)	2 (100)
Cardiovascular system, clinical^d^	10 (100)	3 (100)
Dermatologicals, clinical	1 (100)	1 (100)
Genitourinary system and sex hormones, clinical	2 (100)	2 (100)
Musculoskeletal system		
Clinical	1 (100)	1 (100)
Histologic	(100)	1 (100)
Laboratory	NA	1 (100)
Systemic hormonal preparations, excluding sex hormones and insulins, physiologic	1 (100)	1 (100)
Various		
Clinical	NA	1 (20)
Histologic	2 (14)	NA
Laboratory	12 (86)	3 (60)
Magnetic	2 (14)^e^	2 (40)^e^
Physiologic	NA	1 (20)

Abbreviation: NA, not applicable.

^a^ No preapproval trials have been conducted on humans for the 3 indications for anthrax treatment.

^b^ Study end points were classified as follows: histologic biomarker, laboratory biomarker, magnetic biomarker, physiologic biomarker, and clinical outcome.

^c^ Denominator is the number of confirmatory trials required for each drug indication. Some trials had more than 1 type of end point, in which case the numbers in parentheses add up to more than 100%.

^d^ Indication of treprostinil sodium approved for the treatment of pulmonary arterial hypertension was classified as cardiovascular system instead of blood and blood-forming organs.

^e^ Liver iron concentration determined through the use of a superconducting quantum interference device or magnetic resonance imaging.

### Status of Required Confirmatory Trials

Of the 86 required confirmatory trials, 17 (20%) had not fulfilled postapproval requirements (Table 3). The estimated median time from accelerated approval to confirmatory trial completion was 39.4 (95% CI, 30.7-47.9) months. Results were published in 56 completed confirmatory trials (65%), with the estimated median time from accelerated approval to publication being 52.5 (95% CI, 35.6-82.2) months.

**Table 3.** Regulatory Outcomes of Confirmatory Trials

Outcomes	Trials, No. (%)
Regulatory status	
Fulfilled	69 (80)
Submitted	1 (1)
Ongoing	6 (7)
Delayed	4 (5)
Terminated	2 (2)
Released	2 (2)
Discontinued	1 (1)
Withdrawn	1 (1)
Failure to verify clinical efficacy	9 (10)
Estimated time to postapproval study completion, median (95% CI), mo^a^	39.4 (30.7-47.9)
Published	56 (65)
Estimated time to publication, median (95% CI), mo^a^	52.5 (35.6-82.2)

^a^ Time from the date of accelerated approval.

Nine confirmatory trials (10%), 5 of which had not published results, failed to verify clinical efficacy; 8 drug indications (14%) were associated with these trials (Table 4). Of the 8 drug indications, only 1 (2%; dalfopristin/quinupristin) was withdrawn due to a failed trial 136 months postapproval. Of the remaining 7, 3 led to regular approval conversion with other confirmatory trials demonstrating clinical efficacy. In a confirmatory trial that failed to verify clinical efficacy with protocol violations by many participants, the drug indication (rifapentine for the treatment of pulmonary tuberculosis) was granted regular approval according to equivalence rather than superiority over an active comparator.

**Table 4.** Drug Indications Associated With Confirmatory Trials That Failed to Verify Clinical Efficacy

Drug	Accelerated approval indication	Current FDA status	Trials, no.	Characteristics and outcomes of confirmatory trials						
				Randomization	Blinding	Enrollment, No. of participants	Primary end point	Verification of clinical efficacy	Current FDA status	Publication status
Hydroxy-progesterone caproate	At-risk preterm birth	Not yet converted	1	Randomized	Double-blinded	1740	Time to preterm birth up to 35 wk	Failed	Submitted	Published
							Neonatal morbidity composite at 35 wk			
Dalfopristin/quinupristin	Vancomycin-resistant enterococcus faecium	Withdrawn	Unclear	Unclear	Unclear	Unclear	Unclear	Failed	Withdrawn	Not published
Amprenavir	HIV-1 infection adjunct	Converted to regular approval	2	Randomized	Double-blinded	232	Percentage plasma HIV-1 RNA <400 copies/mL at 48 wk	Verified	Fulfilled	Published
					Open-label	504	Percentage plasma HIV-1 RNA <400 copies/mL at 48 wk	Failed	Fulfilled	Not published
Rifapentine	Pulmonary tuberculosis	Converted to regular approval	1	Randomized	Open-label	722	Percentage negative sputum culture at 24 mo	Failed	Fulfilled	Not published
Mafenide acetate	Bacterial infection in autograft burn dressings	Not yet converted	1	Nonrandomized	Open-label	220	Percentage postautograft graft loss at 5-7 d	Failed	Terminated	Not published
Midodrine hydrochloride	Orthostatic hypotension	Not yet converted	1	Randomized	Double-blinded	67	Response on Orthostatic Hypotension Symptom Assessment (OHSA) change in syncopal/near syncopal events at 16 d	Discontinued	Discontinued	Not published
Interferon β-1b	Ambulatory relapsing-remitting multiple sclerosis	Converted to regular approval	2	Randomized	Double-blinded	718	Disability Progression on Expanded Disability Status Scale (EDSS): 1-point increase or 0.5-point increase for baseline scores ≥6	Verified	Fulfilled	Published
				Randomized	Double-blinded	631	Disability Progression on Expanded Disability Status Scale (EDSS): 1-point increase or 0.5-point increase for baseline scores ≥6	Failed	Fulfilled	Published
Zalcitabine	Advanced and deteriorating HIV infection adjunct	Converted to regular approval	3	Randomized	Double-blinded	1001	Time to development of AIDS	Failed	Fulfilled	Published
							Death			
				Randomized	Double-blinded	2495	Time to ≥50% decrease in CD4 cell count	Verified	Fulfilled	Published
							Development of AIDS			
							Death			
				Randomized	Double-blinded	1113	Time to development of AIDS	Failed	Fulfilled	Published
							Death			

## Discussion

To our knowledge, this cohort study is the most comprehensive study of all nononcology drug indications receiving accelerated approval over the past 26 years. We examined key regulatory consequences of 57 indications with 93 preapproval trials and 86 confirmatory trials. We found that three-quarters of the indications were converted to regular approval. The program expedited drug approval by approximately 4.5 years. However, 1 in 5 confirmatory trials failed to meet FDA requirements. In certain cases, clinical efficacy was unconfirmed. One indication was withdrawn owing to a lack of evidence of efficacy 136 months after accelerated approval. Although there were no safety-related withdrawals, postapproval boxed warnings were often added, indicating that new serious safety risks had been identified after marketing.

According to the FDA’s 2018 review^5^ of 93 oncology indications granted accelerated approval from December 1992 through May 2017, only 55% had fulfilled their postapproval requirements and 5% were withdrawn because of unproven efficacy. In the present study, a higher proportion of nononcology indications eventually converted to regular approval, and fewer were withdrawn owing to the failure of confirmatory trials. This may be attributed in part to the fairly long follow-up periods. This allowed for more event observations. Another important reason may be the fairly inferior designs of oncology drug preapproval trials. Most trials were single-group and response rate was used as a surrogate end point with low or modest thresholds projecting overall survival benefits.^5,28,29^ The ability of response rate as a validated surrogate for overall survival varies across cancer types and is reported to be generally low.^30,31^ In contrast, most preapproval trials of nononcology drugs were randomized clinical trials and the primary end points were diverse, reflecting a wide range of therapeutic areas. The acceptability of surrogate end points is determined case-by-case for each drug indication.^28^ Nevertheless, the same surrogate end point has been used semiuniformly in oncology preapproval trials regardless of cancer type, which may have detrimentally affected the postapproval consequences of oncology drug accelerated approvals.

We found a confirmatory trial with results published more than 10 years after accelerated approval, and others were still ongoing or delayed after more than 8 years. The maximum time from accelerated approval to regular approval exceeded 18 years. In 1 case, the first boxed warning was issued more than 20 years after accelerated approval and the only withdrawal of approval due to a failed confirmatory trial occurred after more than 10 years. These findings suggest that a comprehensive evaluation of drugs may take more than a decade, especially for withdrawal and safety assessments. These findings emphasize the importance of due diligence in conducting confirmatory trials within a reasonable time frame and withdrawing approval immediately if the trial does not demonstrate clinical benefits outweighing the risks. Once granted accelerated approval, delaying further testing could only benefit manufacturers while harming consumers. During the long postmarketing phase, considerable information about drug effectiveness and safety can be discovered through well-controlled observational studies and clinical trials. The FDA and other stakeholders should jointly develop an effective system for prompt postmarketing effectiveness evaluation and safety risk monitoring using clinical practice data, sharing data with independent researchers.

The FDA and other stakeholders are urged to ensure that the provisional nature of approved nononcology drugs is fully communicated to patients, clinicians, and other users, including the fact that a comprehensive risk-benefit assessment can take more than a decade. In particular, patients with illnesses for which there are no treatment options will continue to use expensive drugs with false hope, even in the absence of sufficient evidence of benefit. Drugs given accelerated approval can quickly become a standard of care despite the limited evidence of efficacy.^32^ Clinical guidelines may even continue to recommend drugs with failed confirmatory trials despite withdrawn approval.^33^ Given the recent rising prices of drugs associated with this program, further research is needed to assess the real costs of expediting drug approvals that ultimately prove ineffective or unsafe.

### Limitations

This study has certain limitations. First, as the study sample was limited by the number of accelerated nononcology drug indication approvals, we mainly conducted descriptive analyses. Next, we relied on publicly available information and may have missed some important information despite our rigorous search. In particular, data on label changes that occurred before July 1996 were not well-documented in Drugs@FDA and we relied on manual searches. Additionally, our findings may not be applicable to comparable programs by other regulatory agencies, such as conditional marketing authorization at the European Medicines Agency,^34^ because of different approval processes and timing.^35^ Differences between the FDA and other regulatory agencies in the nononcology area could be an interesting topic for future research.

## Conclusions

Among 57 nononcology indications granted accelerated approval over the past 26 years, 75% were converted to regular approval; however, 20% of confirmatory trials had not fulfilled FDA requirements and clinical efficacy was sometimes unconfirmed. Furthermore, additional boxed warnings were often issued after accelerated approval. Thus, the benefits and risks of nononcology drugs receiving accelerated approval were provisional and their comprehensive evaluation will take more than a decade. Our findings underscore the importance for the FDA and other stakeholders to maintain and increase vigilance over accelerated approval of nononcology drugs as well as oncology drugs.

## Notes

Supplement. **eFigure.** Flowchart Showing the Selection of Drug Indications Approved by the FDA, June 1992 to May 2018 **eTable.** Nononcology Drugs and FDA Accelerated Approval Indications, June 1992 to May 2018
