# Supplementary material for: US Food and Drug Administration Accelerated Approval Program for Nononcology Drug Indications Between 1992 and 2018
Source: JAMA Netw Open. 2022 Sep 9;5(9):e2230973. doi: 10.1001/jamanetworkopen.2022.30973 (PMC9463606; doi:10.1001/jamanetworkopen.2022.30973)
Supplement: Supplement. — eFigure. Flowchart Showing the Selection of Drug Indications Approved by the FDA, June 1992 to May 2018 eTable. Nononcology Drugs and FDA Accelerated Approval Indications, June 1992 to May 2018 [file jamanetwopen-e2230973-s001.pdf]

Supplemental Online Content

Omae K, Onishi A, Sahker E, Furukawa TA. US Food and Drug Administration accelerated approval program for nononcology drug indications between 1992 and 2018. *JAMA Netw Open*. 2022;5(9):e2230973. doi:10.1001/jamanetworkopen.2022.30973

eFigure. Flowchart Showing the Selection of Drug Indications Approved by the FDA, June 1992 to May 2018
eTable. Nononcology Drugs and FDA Accelerated Approval Indications, June 1992 to May 2018

This supplemental material has been provided by the authors to give readers additional information about their work.

© 2022 Omae K et al. *JAMA Network Open*.

eFigure. Flowchart Showing the Selection of Drug Indications Approved by the FDA, June 1992 to May 2018

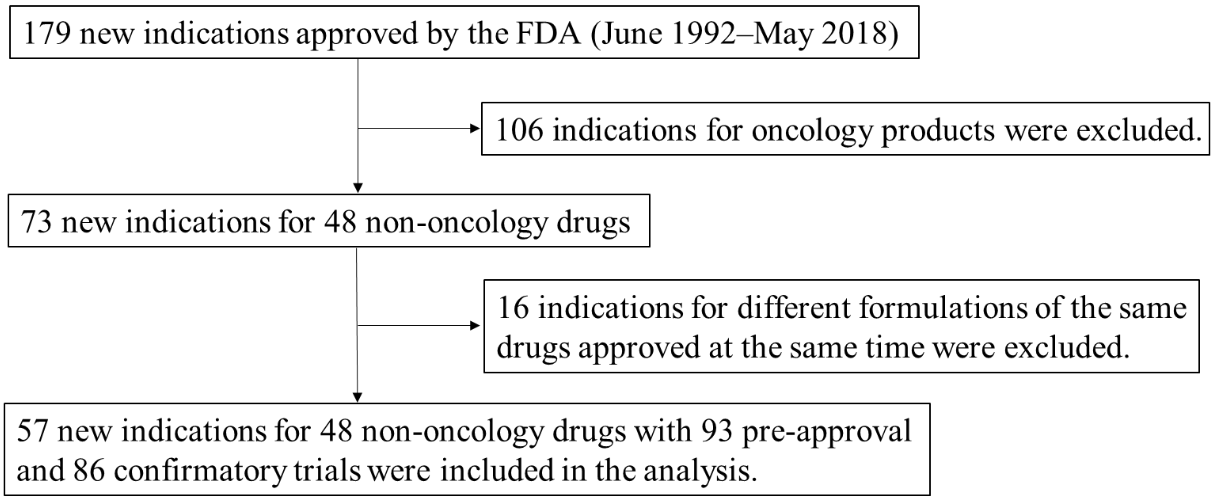

© 2022 Omae K et al. *JAMA Network Open*.

eTable. Nononcology Drugs and FDA Accelerated Approval Indications, June 1992 to May 2018

Drug	Approval Date	Accelerated Approval Indication	Therapeutic Area[a]	Novel or Supplemental Indications	Current FDA Status
Benznidazole	2017/8/29	Chagas disease (American trypanosomiasis caused by Trypanosoma cruzi) – ages: 2-12	Antiparasitic products, insecticides and repellents	Novel	Not yet converted
Deferasirox	2017/5/18	Transfusional chronic iron overload – ages: 2+	Various	Novel	Converted to regular approval
Deferasirox	2017/5/18	Non-transfusion-dependent thalassemia Chronic iron overload – ages:10+	Various	Novel	Converted to regular approval
Eteplirsen	2016/9/19	Duchenne Muscular Dystrophy (DMD)	Musculo-skeletal system	Novel	Not yet converted
Obeticholic acid	2016/5/27	Primary biliary cholangitis combined with ursodeoxycholic acid (UDCA)	Alimentary tract and metabolism	Novel	Not yet converted
Methylene blue	2016/4/8	Acquired methemoglobinemia	Various	Novel	Not yet converted
Idarucizumab	2015/10/16	Pradaxa® patients needing reversal of the anticoagulant effects and general uncontrolled bleeding	Various	Novel	Converted to regular approval

© 2022 Omae K et al. *JAMA Network Open.*

Drug	Approval Date	Accelerated Approval Indication	Therapeutic Area[a]	Novel or Supplemental Indications	Current FDA Status
Deferiprone	2015/9/9	Thalassemia syndromic transfusional iron overload due to inadequate chelation therapy	Various	Novel	Converted to regular approval
Deferasirox	2015/3/30	Transfusional chronic iron overload – ages: 2+	Various	Novel	Converted to regular approval
Deferasirox	2015/3/30	Non-transfusion-dependent thalassemia Chronic iron overload – ages:10+	Various	Novel	Converted to regular approval
Droxidopa	2014/2/18	Neurologic, neuropathic, or dopaminergic orthostatic dizziness	Cardiovascular system	Novel	Not yet converted
Idursulfase	2013/6/24	Hunter syndrome adjunctive – ages: 5+	Alimentary tract and metabolism	Supplemental	Not yet converted
Deferasirox	2013/1/23	Non-transfusion-dependent thalassemia Chronic iron overload – ages:10+	Various	Supplemental	Converted to regular approval
Bedaquiline	2012/12/28	Pulmonary multi-drug resistant tuberculosis adjunctive – ages: 18+	Anti-infectives for systemic use	Novel	Not yet converted

© 2022 Omae K et al. *JAMA Network Open*.

Drug	Approval Date	Accelerated Approval Indication	Therapeutic Area[a]	Novel or Supplemental Indications	Current FDA Status
Deferiprone	2011/10/14	Thalassemia syndromic transfusional iron overload due to inadequate chelation therapy	Various	Novel	Converted to regular approval
Hydroxyprogesterone caproate	2011/2/3	At-risk preterm birth	Genitourinary system and sex hormones	Novel	Not yet converted
Eltrombopag	2008/11/20	Chronic immune thrombocytopenia with insufficient response to corticosteroids, immunoglobulins, or splenectomy	Blood and blood forming organs	Novel	Converted to regular approval
Levofloxacin	2008/5/5	Exposure to aerosolized bacillus anthracis prophylaxis – ages: 6 months+	Anti-infectives for systemic use	Supplemental	Withdrawn
Etravirine	2008/1/18	Treatment-experienced HIV-1 infection with viral replications and HIV-1 antiretroviral resistant adjunctive – ages: 18+	Anti-infectives for systemic use	Novel	Converted to regular approval

© 2022 Omae K et al. *JAMA Network Open*.

Drug	Approval Date	Accelerated Approval Indication	Therapeutic Area[a]	Novel or Supplemental Indications	Current FDA Status
Raltegravir	2007/10/12	Treatment-experienced HIV-1 infection with viral replications and HIV-1 antiretroviral resistant (multiple trials) adjunctive – ages: 18+	Anti-infectives for systemic use	Novel	Converted to regular approval
Maraviroc	2007/8/6	CCR5-tropic HIV-1	Anti-infectives for systemic use	Novel	Converted to regular approval
Darunavir	2006/6/23	Treatment-experienced HIV infection with HIV-1 strains resistant to more than one protease inhibitor – ages: 18+	Anti-infectives for systemic use	Novel	Converted to regular approval
Deferasirox	2005/11/2	Transfusional chronic iron overload – ages: 2+	Various	Novel	Converted to regular approval
Tipranavir	2005/6/22	HIV-1 with viral replication, treatment experience, or multiple protease inhibitor resistance - ritonavir 200 mg adjunct	Anti-infectives for systemic use	Novel	Converted to regular approval
Levofloxacin	2004/11/24	Inhalational anthrax	Anti-infectives for systemic use	Supplemental	Withdrawn

© 2022 Omae K et al. *JAMA Network Open*.

Drug	Approval Date	Accelerated Approval Indication	Therapeutic Area[a]	Novel or Supplemental Indications	Current FDA Status
Treprostinil sodium	2004/11/24	Pulmonary arterial hypertension - treprostinil sodium 1, 2.5, 5 & 10 mg/ml injection adjunct	Cardiovascular system[b]	Supplemental	Converted to regular approval
Natalizumab	2004/11/23	Relapsing multiple sclerosis	Antineoplastic and immunomodulating agents	Novel	Converted to regular approval
Lutropin alpha	2004/10/8	Follicular stimulation in hypogonadotropic hypogonadal women with profound LH deficiency - Gonal-f adjunct	Genitourinary system and sex hormones	Novel	Withdrawn
Emtricitabine and tenofovir disoproxil fumarate	2004/8/2	HIV infection adjunctive – ages: 18+	Anti-infectives for systemic use	Novel	Converted to regular approval
Agalsidase beta	2003/4/24	Fabry disease	Alimentary tract and metabolism	Novel	Converted to regular approval
Enfuvirtide	2003/3/13	HIV-1 infection adjunct in treatment-experienced patients with HIV-1 replication despite ongoing antiretroviral therapy	Anti-infectives for systemic use	Novel	Converted to regular approval

© 2022 Omae K et al. *JAMA Network Open*.

Drug	Approval Date	Accelerated Approval Indication	Therapeutic Area[a]	Novel or Supplemental Indications	Current FDA Status
Treprostinil sodium	2002/5/21	Pulmonary arterial hypertension	Cardiovascular system[b]	Novel	Converted to regular approval
Tenofovir disoproxil fumarate	2001/10/26	HIV-1 infection – ages: 18+	Anti-infectives for systemic use	Novel	Converted to regular approval
Abacavir sulfate; lamivudine; zidovudine	2000/11/14	HIV-1 infection	Anti-infectives for systemic use	Novel	Converted to regular approval
Lopinavir: ritonavir	2000/9/15	HIV-1 infection adjunct – ages: 6 months+	Anti-infectives for systemic use	Novel	Converted to regular approval
Ciprofloxacin	2000/8/30	Inhalational anthrax	Anti-infectives for systemic use	Supplemental	Converted to regular approval
Dalfopristin/quinupristin	1999/9/21	Vancomycin-resistant enterococcus faecium	Anti-infectives for systemic use	Novel	Withdrawn
Amprenavir	1999/4/15	HIV-1 infection adjunct	Anti-infectives for systemic use	Novel	Converted to regular approval
Abacavir sulfate	1998/12/17	HIV-1 infection adjunct	Anti-infectives for systemic use	Novel	Converted to regular approval

© 2022 Omae K et al. *JAMA Network Open*.

Drug	Approval Date	Accelerated Approval Indication	Therapeutic Area[a]	Novel or Supplemental Indications	Current FDA Status
Efavirenz	1998/9/17	HIV-1 infection adjunct	Anti-infectives for systemic use	Novel	Converted to regular approval
Nevirapine	1998/9/11	HIV-1 infection adjunct	Anti-infectives for systemic use	Novel	Converted to regular approval
Infliximab	1998/8/24	Moderately to severe Crohn's disease with inadequate response to conventional therapies and fistulizing Crohn's disease	Antineoplastic and immunomodulating agents	Novel	Converted to regular approval
Rifapentine	1998/6/22	Pulmonary tuberculosis	Anti-infectives for systemic use	Novel	Converted to regular approval
Mafenide acetate	1998/6/5	Excised burn wound bacterial infection in meshed autografts adjunct	Dermatologicals	Novel	Not yet converted
Delavirdine mesylate	1997/4/4	HIV-1 infection adjunct	Anti-infectives for systemic use	Novel	Converted to regular approval
Nelfinavir mesylate	1997/3/14	HIV infection	Anti-infectives for systemic use	Novel	Converted to regular approval

© 2022 Omae K et al. *JAMA Network Open*.

Drug	Approval Date	Accelerated Approval Indication	Therapeutic Area[a]	Novel or Supplemental Indications	Current FDA Status
Midodrine hydrochloride	1996/9/6	Symptomatic orthostatic hypotension	Cardiovascular system	Novel	Not yet converted
Somatropin	1996/8/23	AIDS wasting and cachexia	Systemic hormonal preparations, excl. Sex hormones and insulins	Novel	Converted to regular approval
Nevirapine	1996/6/21	Deteriorating HIV-1 infection adjunctive with nucleoside analogues	Anti-infectives for systemic use	Novel	Converted to regular approval
Indinavir sulfate	1996/3/13	HIV-1 infection – ages 18+	Anti-infectives for systemic use	Novel	Converted to regular approval
Ritonavir	1996/3/1	HIV-1 infection adjunct with nucleoside analogs or as monotherapy	Anti-infectives for systemic use	Novel	Converted to regular approval
Saquinavir mesylate	1995/12/6	HIV-1 infection adjunct with nucleoside analogs	Anti-infectives for systemic use	Novel	Converted to regular approval
Lamivudine	1995/11/17	HIV infection adjunct with retrovir (zidovudine)	Anti-infectives for systemic use	Novel	Converted to regular approval

© 2022 Omae K et al. *JAMA Network Open*.

Drug	Approval Date	Accelerated Approval Indication	Therapeutic Area[a]	Novel or Supplemental Indications	Current FDA Status
Stavudine	1994/6/24	Advanced HIV infection for those intolerant to approved therapies	Anti-infectives for systemic use	Novel	Converted to regular approval
Clarithromycin	1993/12/23	Disseminated mycobacterial infections due to mycobacterium avium and mycobacterium intracellulare	Anti-infectives for systemic use	Novel	Converted to regular approval
Interferon beta-1b	1993/7/23	Relapsing-remitting multiple sclerosis	Antineoplastic and immunomodulating agents	Novel	Converted to regular approval
Zalcitabine	1992/6/19	Advanced HIV infection zidovudine adjunct – ages: 18+	Anti-infectives for systemic use	Novel	Converted to regular approval

[a]Therapeutic areas were classified according to the Anatomical Therapeutic Chemical (ATC) classification system index 2022.

[b]Indication of treprostinil sodium approved for the treatment of pulmonary arterial hypertension was classified as "cardiovascular system" instead of "blood and blood forming organs".

© 2022 Omae K et al. *JAMA Network Open*.
